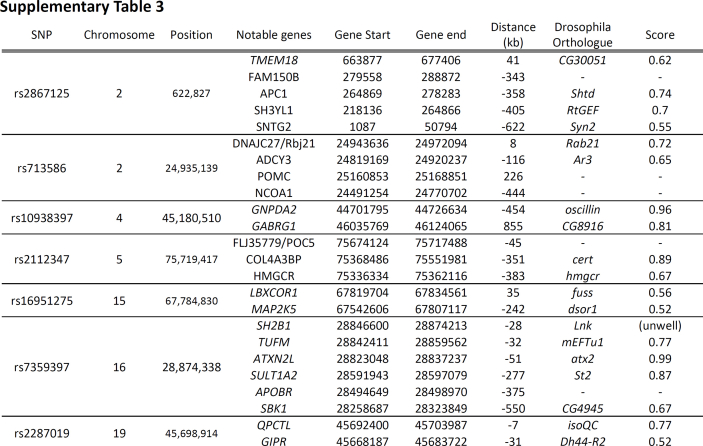# A multicomponent screen for feeding behaviour and nutritional status in *Drosophila* to interrogate mammalian appetite-related genes

**DOI:** 10.1016/j.molmet.2020.101127

**Published:** 2020-11-23

**Authors:** J. Chalmers, Y.C.L. Tung, C.H. Liu, C.J. O'Kane, S. O'Rahilly, G.S.H. Yeo

**Affiliations:** 1 Medical Research Council (MRC) Metabolic Diseases Unit, University of Cambridge Metabolic Research Laboratories, Wellcome Trust–MRC Institute of Metabolic Science, Addenbrooke's Hospital, Cambridge, UK; 2 Department of Genetics, University of Cambridge, Downing Street, Cambridge, CB2 3EH, UK; 3 Department of Physiology, Development and Neuroscience, Cambridge University, Downing St, Cambridge, CB2 3EG, UK

**Keywords:** Appetite, Food intake, GWAS, Transcriptomics, *Drosophila*, Obesity

## Abstract

**Objective:**

More than 300 genetic variants have been robustly associated with measures of human adiposity. Highly penetrant mutations causing human obesity do so largely by disrupting satiety pathways in the brain and increasing food intake. Most of the common obesity-predisposing variants are in, or near, genes expressed highly in the brain, but little is known of their function. Exploring the biology of these genes at scale in mammalian systems is challenging. We sought to establish and validate the use of a multicomponent screen for feeding behaviour phenotypes, taking advantage of the tractable model organism *Drosophila melanogaster*.

**Methods:**

We validated a screen for feeding behaviour in *Drosophila* by comparing results after disrupting the expression of centrally expressed genes that influence energy balance in flies to those of 10 control genes. We then used this screen to explore the effects of disrupted expression of genes either a) implicated in energy homeostasis through human genome-wide association studies (GWAS) or b) expressed and nutritionally responsive in specific populations of hypothalamic neurons with a known role in feeding/fasting.

**Results:**

Using data from the validation study to classify responses, we studied 53 *Drosophila* orthologues of genes implicated by human GWAS in body mass index and found that 15 significantly influenced feeding behaviour or energy homeostasis in the *Drosophila* screen. We then studied 50 *Drosophila* homologues of 47 murine genes reciprocally nutritionally regulated in POMC and agouti-related peptide neurons. Seven of these 50 genes were found by our screen to influence feeding behaviour in flies.

**Conclusion:**

We demonstrated the utility of *Drosophila* as a tractable model organism in a high-throughput genetic screen for food intake phenotypes. This simple, cost-efficient strategy is ideal for high-throughput interrogation of genes implicated in feeding behaviour and obesity in mammals and will facilitate the process of reaching a functional understanding of obesity pathogenesis.

## Highlights

• High-throughput technologies have generated many candidate genes with a potential role in the control of food intake and body weight.
• Gaining an understanding of appetite requires exploration in the context of a whole animal, but studies in humans and mice require a long duration and are expensive.
• The fruit fly is evolutionarily distant but has conserved neuronal circuitry involved in the control of food intake.
• Use of fruit flies is time and cost efficient; therefore, *Drosophila* represent a useful model system for screening candidate genes implicated in feeding behaviour and obesity in mammals.

## 1 Introduction

Obesity is arguably the most serious public health threat of the 21st century [1] because of its association with comorbidities such as type 2 diabetes, cardiovascular disease, hypertension, and certain cancers [2]. Modern lifestyles have been the driver of the increase in obesity, and the variation in individuals' response to this ‘obesogenic’ environment is large [3]. Underlying this variable response is a powerful genetic element: twin and adoption studies have revealed the heritability of fat mass to be between 40% and 70% [4,5].

Over the past 20 years, genetic and ‘omics’ approaches have been used to characterise the molecular and physiological mechanisms of food intake control. For instance, studies of human and mouse genetics have uncovered circuits within the brain that play a central role in modulating mammalian appetitive behaviour [6,7]. The best characterised of these circuits is the hypothalamic leptin-melanocortin signalling pathway, genetic disruption of which causes the majority of monogenic severe obesity disorders in mice and humans [[8], [9], [10], [11]]. In addition, genome-wide association studies (GWAS) have identified more than 300 human genetic loci associated with variations in body mass index (BMI) [12,13]. The genes closest to these loci, including many components of the melanocortin pathway, are primarily expressed in the central nervous system (CNS) [13,14]. Where their function is known, many of these genes influence food intake [[6], [7], [8], [9], [10], [11],[15], [16], [17], [18], [19]].

In addition to genetic approaches, transcriptomic analyses of discrete neuronal populations are playing an increasingly important role in illuminating novel genes and pathways that may play a role in appetite control [20,21]. At least 2 populations of neurons sense peripheral nutritional signals and play a central role in the melanocortin pathway: pro-opiomelanocortin (POMC) neurons decrease food intake when activated, and agouti-related peptide (AgRP) neurons increase food intake [22,23]. Some transcripts are reciprocally regulated in these 2 populations [21], but the function of these transcripts remains mostly unknown.

One reason for the disappointing rate of translating the genetic signals into insightful biological knowledge is that investigations of candidate genes have been mainly addressed in complex model organisms such as mice [[24], [25], [26], [27], [28], [29], [30], [31], [32]]. Given the significant resources required to generate and phenotype each murine model, they are not ideal for studying the effects of disruption of gene function at scale. Thus, a high-throughput model is necessary to ‘pre-screen’ genes for a potential role in feeding behaviour, before committing the resources necessary for further research in mammalian models.

The fruit fly *Drosophila melanogaster* is a key model organism for research in developmental biology, cell biology, and neurobiology; has recently been demonstrated as an excellent model for dissecting metabolic homeostasis and nutrient-sensing pathways; and has a rapid generation time of 10 days, high fecundity (up to 100 eggs produced per female fly per day [33]), and a 10,000-fold lower cost to maintain than mice [34]. In addition, mutant fly lines and other resources are abundant and freely available within the scientific community [34]. Although some mammalian biology is not replicated, ∼75% of genes involved in inherited human diseases have an orthologue in flies [35,36]. Furthermore, there is substantial conservation of tissue-specific patterns of gene expression, suggesting that high levels of functional conservation are also likely [36].

Here, we report a high-throughput *in vivo* functional screen for genes involved in mammalian feeding behaviour and nutritional status using *Drosophila*. Because feeding behaviour is largely controlled by the brain, we use a neuron-specific approach involving RNA interference (RNAi) knockdown. We validated a suite of 3 assays by their ability to detect nutritional perturbation in wild-type flies and to distinguish between positive and negative control genes. We then used these assays to screen 2 datasets. First, we studied 53 orthologues of human genes near to single nucleotide polymorphisms (SNPs) identified by GWAS to be robustly associated with human adiposity. In addition, we studied 50 *Drosophila* genes corresponding to 47 murine genes that were reciprocally regulated in mouse POMC and AgRP neurons in response to an overnight fast. With both datasets, we demonstrated the utility of a tractable model organism in a high-throughput genetic screen for food intake phenotypes and identified candidates to pursue in further research.

## 2 Materials and methods

### 2.1 Fly husbandry

All flies were raised on a normal diet (ND: 1.25% agar, 10.5% dextrose, 9% maize, 2.6% yeast, 3.5% nipagin in water). Some assays required the use of food with added dye (ND + 1% Fast Green FCF dye [Sigma]) or a high-fat diet (HFD: ND + 20% coconut oil). Experimental flies were maintained at 25 °C and 60%–70% humidity in a 14-hour light: 10-hour dark cycle, except for flies on the HFD, which were housed at 20 °C in the dark. Unless otherwise noted, we used male flies aged between 5 and 10 days for the experiments, 15 flies per sample, and 5 biological replicates per genotype per assay.

*Drosophila* orthologues of human genes of interest were identified by using ENSEMBL [37] or NCBI BLAST searches of publicly available protein sequences [38,39]. Fly stocks (listed in Supplementary Table 1) were acquired from the Vienna Drosophila Resource Centre (VDRC) and the Bloomington Drosophila Stock Centre. For RNAi stocks, preference was given to GD lines to avoid potential complications from *tiptop* gene expression [40]. Two *GAL4* lines were used: *elav-GAL4/CyO*, expressed in neurons [41], and *act5C-GAL4/CyO*, constitutively expressed throughout the body. *UAS-RNAi* lines were crossed with *Gal4* lines (*elav-Gal4* or *act-Gal4*), and controls were created by crossing each Gal4 line with control lines for *GD*, *KK*, and *KK*_*tiptop*_. Experimental flies were compared with the relevant background control (Supplementary Table 1). Three of the *GD* lines (*ADCY3*/*Ac3*, *LRRN6C*/*Fili*, and *NEGR1*/*Ama*) were supplied as compound *X* stocks from VDRC and were rebalanced by using FM7 before phenotyping.

To standardise the effects of the parental environment on offspring fitness, all *UAS-RNAi* stocks were stored in bottles at approximately constant density. Flies for phenotyping were generated by crossing virgin female *UAS-RNAi* flies to male *GAL4* flies and collecting the male offspring. To standardise the effects of parental age on offspring fitness, crosses were set up by using 1–5-day-old flies. Briefly, for each cross, on day 1, 5 *UAS-RNAi* female virgins were placed in a vial of ND at 25 °C with 2 *elav-Gal4* or 5 *act-Gal4* males. On day 4, these parents were removed. On day 14, offspring were transferred to a new vial and allowed to mate because mating alters gene expression and metabolic parameters [42]. On day 15, the females were removed. On day 16 at 2 pm, male flies were placed on starvation media for 3 h to synchronise their metabolism before being returned to the appropriate diet for the experiment. Because *Drosophila* activity and feeding behaviours are affected by age and circadian rhythms [43], all assays were performed within a fixed time window: the capillary feeder (CAFE) assay was performed at 2 pm; the fasting-induced overfeeding assay, at 9 am; and sample collection for wet mass and triglyceride analysis, at 10 am.

### 2.2 PCR-based diagnostic assay to determine appropriate background line for the ‘KK’ collections

Genomic DNA was isolated by crushing flies in ‘squishing buffer’ (10 mM Tris, pH8.2, 1 mM EDTA, 25 mM NaCl, 200 μg/ml protease K), followed by a 30-minute incubation at 37 °C, after which the proteinase K was inactivated by heating at 95 °C for 5 min. We used a PCR-based diagnostic assay to determine the *KK* insertion site, as described in Green et al. [40]. Briefly, occupancy of the transgene at the annotated insertion site reported by the VDRC, *40D*, was determined in a multiplex PCR. The following primers and PCR using these primers yield a ∼450-bp product in the case of an insertion, or a ∼1050-bp product in the case of an empty insertion site, at *40D*:

40D Genomic_F 5′- GCCCACTGTCAGCTCTCAAC -3′
pKC26_R 5′- TGTAAAACGACGGCCAGT -3′
pKC43_R 5′- TCGCTCGTTGCAGAATAGTCC-3′

For detecting the non-annotated insertion, *30B* (thus we used *tiptop* as control), the pKC26_R and pKC43_R primers were multiplexed with the following primer:

30B_Genomic_F 5′- GCTGGCGAACTGTCAATCAC -3′

PCR using these primers results in a ∼600-bp product in the case of an insertion, and a ∼1200-bp product in the case of an empty insertion site, at *30B*.

PCR was performed with the following programme: 95 °C 120 s initial denaturation, 30 cycles (95 °C 15 s denaturation, 50 °C 15 s annealing, 72 °C 45 s extension), and a final 72 °C 120s extension.

### 2.3 CAFE assay

**Fig S1:**
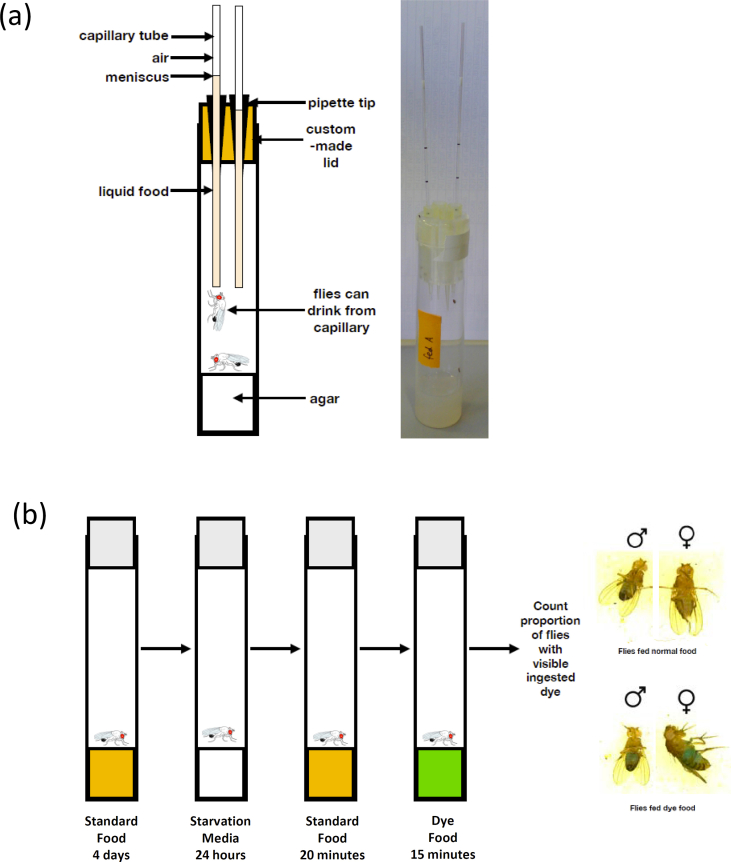


The CAFE assay is an accurate method of measuring food intake in *Drosophila* [44] for which the liquid food in calibrated capillary glass tubes (5 μl, VWR International) is the only food available to the study flies. The CAFE assay was performed using a custom-made acrylic cap with 4 holes that hold 200 μl pipette tips; of the 4 holes, 2 act as air holes and 2 hold the glass capillaries (VWR 53432-706) in place. CAFE chambers were made from standard fly vials (25 × 95 mm) with 3 ml of 1% agar that serves as a water source and maintains internal chamber humidity (Supplementary Figure 1). Eight flies were placed into each vial. Two capillary tubes were filled with liquid food (5% sucrose and 5% yeast extract) via capillary action. The top of the meniscus was marked, and the filled capillaries were inserted into the chamber through the lid and left at 25 °C. After an initial 48 h of habituation in the CAFE setup, the movement of the meniscus was measured over 24 h and evaporation (measured by a vial containing no flies) was subtracted to yield the volume of food consumed in 24 h.

### 2.4 Fasting-induced overfeeding assay

This protocol was modified from Williams et al., 2014 [45] and is particularly useful for examining satiety signals. Fifteen male flies were fasted for 24 h in vials with 1% agar. On the day of the experiment, these flies were transferred to ND and allowed to feed. After 20 min, the flies were then transferred to vials containing ND with 1% Fast Green dye for 15 min (Supplementary Figure 1). The number of flies with visible dye in their abdomen (mid-gut and/or crop) were counted under a dissecting microscope and scored as a percentage of the total number in the vial.

### 2.5 Wet mass

Groups of 15 *Drosophila* were frozen on dry ice and weighed by using a microbalance (Sartorius) rounded to 2 decimal places in milligrams.

### 2.6 Triglyceride assays

For triglyceride analysis, frozen *Drosophila* were placed in FastPrep tubes containing Lysis Beads and Matrix D (MP Biomedicals) and 350 μl cold PBST (PBS + 0.05% Tween-20) and then homogenised by using a FastPrep-24 homogeniser (MP Biomedicals) for 60 s at 6 m/s. Solutions were centrifuged (16,100 rcf, 4 °C, 3 min) to pellet debris, and 300 μl of supernatant pipetted into a fresh Eppendorf on ice. Homogenates were heat-inactivated (5 min, 70 °C). Triglyceride levels were analysed by using enzymatic assays by the Cambridge Core Biochemical Assay Laboratory. Triglyceride amount was normalised to the number of *Drosophila*.

### 2.7 Phenotyping of larvae

Cages were set up containing 60 virgin female and 30 male flies on apple juice agar plates with fresh yeast paste and left for 3 days at 25 °C. On the day of collection, a new plate with fresh yeast paste was provided every 30 min for 1 h to clear old embryos from the oviducts of female flies. Embryos were collected on a fresh plate for 3 h and then left for 72 h to develop into 3rd instar larvae. Larvae were either fed liquid yeast or fasted (provided with distilled water instead) for 2 h. For the assay, yeast paste was placed in the centre of an apple juice agar plate, and 20 larvae were placed on the inside rim. The number of larvae in/out of the food was counted after 20 min.

### 2.8 Scoring algorithm

The scoring system ‘sums up’ data for all 3 assays performed in adult flies to provide a quantitative measure of the overall phenotype. The scoring sums the p values for each assay and then subtracts from 1 so that more significant results yield higher scores. We used at least 5 sets of biological replicates for each assays and 15 flies per assay (except for the CAFE assay, which had 8 flies per assay).

### 2.9 Statistical analysis

Each assay was repeated at least 5 times by using independent crosses (biological replicates). Mean and standard error of the mean were calculated from all biological replicates of each experiment and analysed by using GraphPad Prism. Data from all assays were analysed using unpaired homoscedastic Student t tests. ANOVA with appropriate *post hoc* analysis for multiple comparisons was employed where appropriate.

## 3 Results

### 3.1 Selection of assays for the high-throughput screen

**Figure 1:**
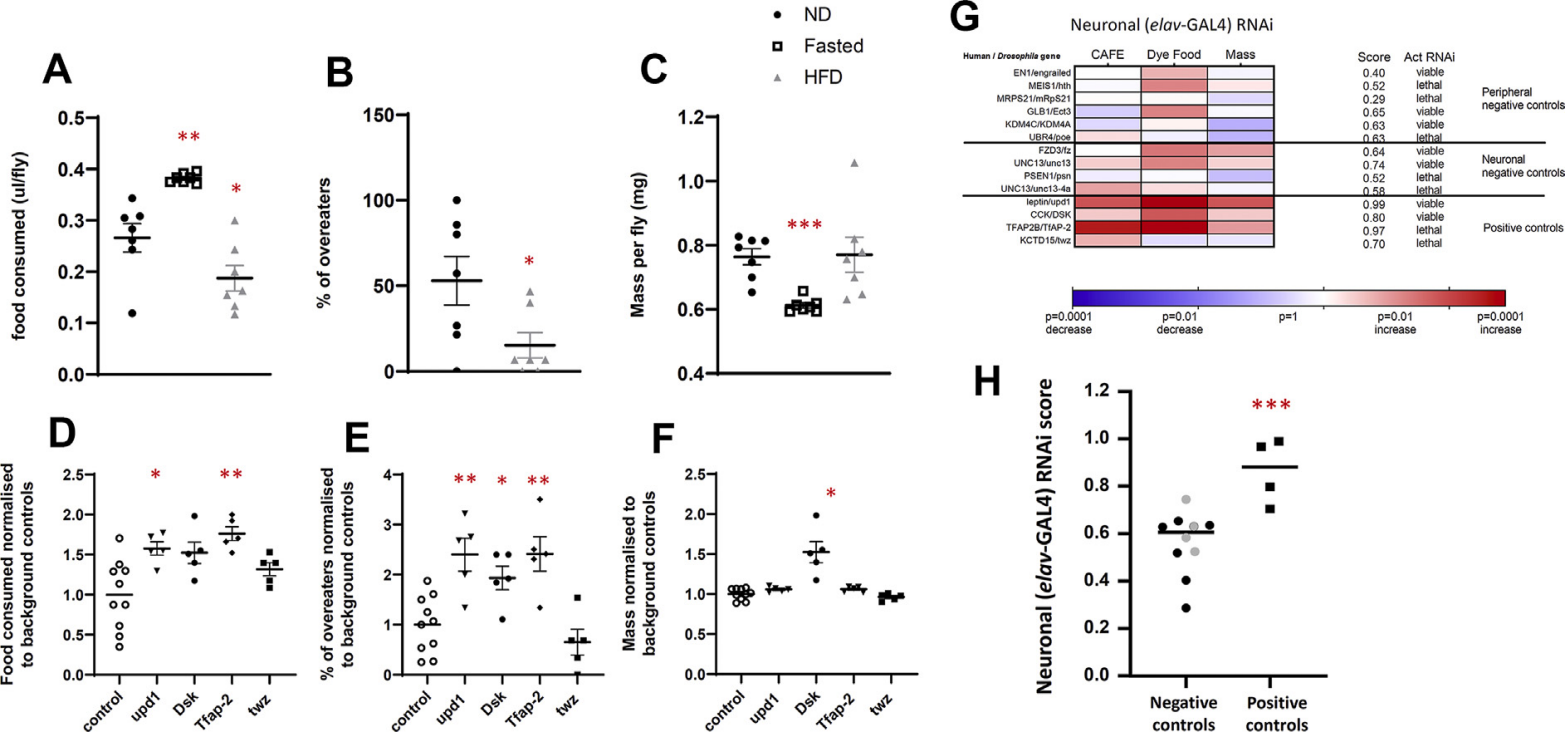
Validation of assays used in the screen.

To create a *Drosophila*-based functional screen focused on appetitive behaviour, we selected 3 assays: body mass and 2 measures of energy intake (the CAFE assay and the fasting-induced feeding assay). To validate these assays, we tested each for their ability to differentiate wild-type flies on a ND from those either fasted for 24 h or placed on a HFD for 5 days (Figure 1).

The CAFE assay measures the amount of liquid food (sucrose plus yeast extract) that flies consume from a capillary tube by tracking the distance moved by the meniscus over a given time. As expected, fasted flies consumed significantly more than the controls (144%; p = 0.0012) did, and flies on an HFD consumed less (70%; p = 0.0538; Figure 1A).

The fasting-induced feeding assay measures food intake after fasting. Flies were fasted for 24 h and then exposed to normal food for 20 min before being transferred onto food coloured with dye for 15 min, which is then visible through their translucent abdomen. In the HFD group, the number of flies with green stomachs decreased to 29% of that of controls (Figure 1B, p = 0.036).

Fasted flies, as expected, had less wet mass (80%, p < 0.0001) than flies fed *ad libitum*, and a HFD had no measurable effect on mass (Figure 1C).

Therefore, the 3 assays detected physiologically meaningful responses of wild-type flies to nutritional perturbation.

### 3.2 Validation of the high-throughput screen using ‘control’ genes

Next, we tested our screen on 4 genes that play roles in *Drosophila* energy homeostasis (Table 1a). Because most genes linked to BMI are enriched for expression or function in the CNS [13], we took advantage of the *UAS*_*G*_*-GAL4* system [46] to knock down the expression of each gene specifically in neurons by using the neuronal driver *elav*-*GAL4* [41]. Results from the neuronal knockdown were compared with background-matched control lines (*KK-elavGAL4*, *GD-elavGAL4*, or *KK*_*tiptop*_*-elavGAL4* as appropriate; Supplementary Table 1). As one example, neuronal knockdown of *upd1*, a *Drosophila* orthologue of leptin [47], led to flies ingesting 1.5× more in the CAFE assay than in controls (p = 0.0149; Figure 1D and 2.4x more flies ingesting dyed food in the fasting-induced feeding assay (p = 0.0013; Figure 1E).

**Table 1:** Positive and negative control genes. Positive and negative control genes were selected from the literature. (a) The positive control genes are involved in the neuronal control of feeding in mammals and all show a phenotype in at least one of the screen assays. (b) For the negative controls, some have a neuronal aetiology (top) and others have no neuronal association (bottom); for all, the primary symptoms are non-metabolic.

Human Gene	Drosophila gene	% Identity	Note
leptin (47)	upd1	6	Functional Orthologue
CCK (48)	DSK	14	Functional Orthologue
TFAP2B (45)	Tfap2	40	
KCTD15 (45)	twz	36	


Disease	Body weight association	Human gene	Drosophila gene	% Identity
Schizophrenia	Obesity (65)	FZD3 (71)	fz	42
ALS	Weight loss (63)	UNC13 (72)	unc13	56
ALS	Weight loss (63)	UNC13 (72)	unc13-4a	56
Alzheimer's	Weight loss (66)	PSEN1 (70)	psn	51

Disease	Patients' experience	Human gene	Drosophila gene	% Identity
Osteoporosis	Anorexic patients (62)	EN1 (77)	en	36
RLS	High adiposity (68)	MEIS1 (76)	hth	60
Atopic dermatitis	Overweight (67)	MRPS21 (75)	mRpS21	49
Allergic Disease	High BMI (64)	GLB1 (73)	ect3	43
HIV infection	–	KDM4C (74)	kdm4a	28
HIV infection	–	UBR4 (74)	poe	35

To take advantage of the multiple assays, we used a simple algorithm to produce an integrated score that reflects the overall phenotype resulting from neuronal knockdown of each gene. The score was calculated by taking the sum of the p values from the 3 assays for each gene and subtracting it from 1. Thus, the lower the p value, the more significant the results for a given gene, and the closer the score would be to 1. Using this method produced an integrated score of 0.99 for *upd1* (Figure 1G). The scores for the other positive controls were 0.80 for *DSK*; 0.97 for *Tfap2*; and 0.70 for the *twz* (the *Drosophila* orthologues of *CCK* [48], *TfAP-2*, and *KCTD15,* respectively [45].

In addition, we tested 10 negative control genes linked to diseases other than obesity (Table 1b). When the scores for all of the controls were plotted, the flies with neuronal knockdown of positive control genes had a significantly higher average score than the negative controls (p = 0.0006; Figure 1H). There was overlap between the scores for peripheral negative and positive controls, but on the basis of its ability to distinguish these 2 groups, we set a score of 0.80 as a threshold for the selection of genes of interest, with consideration given to genes with a score ≥0.70.

### 3.3 Screening of candidate genes from GWAS for BMI

**Figure 2:**
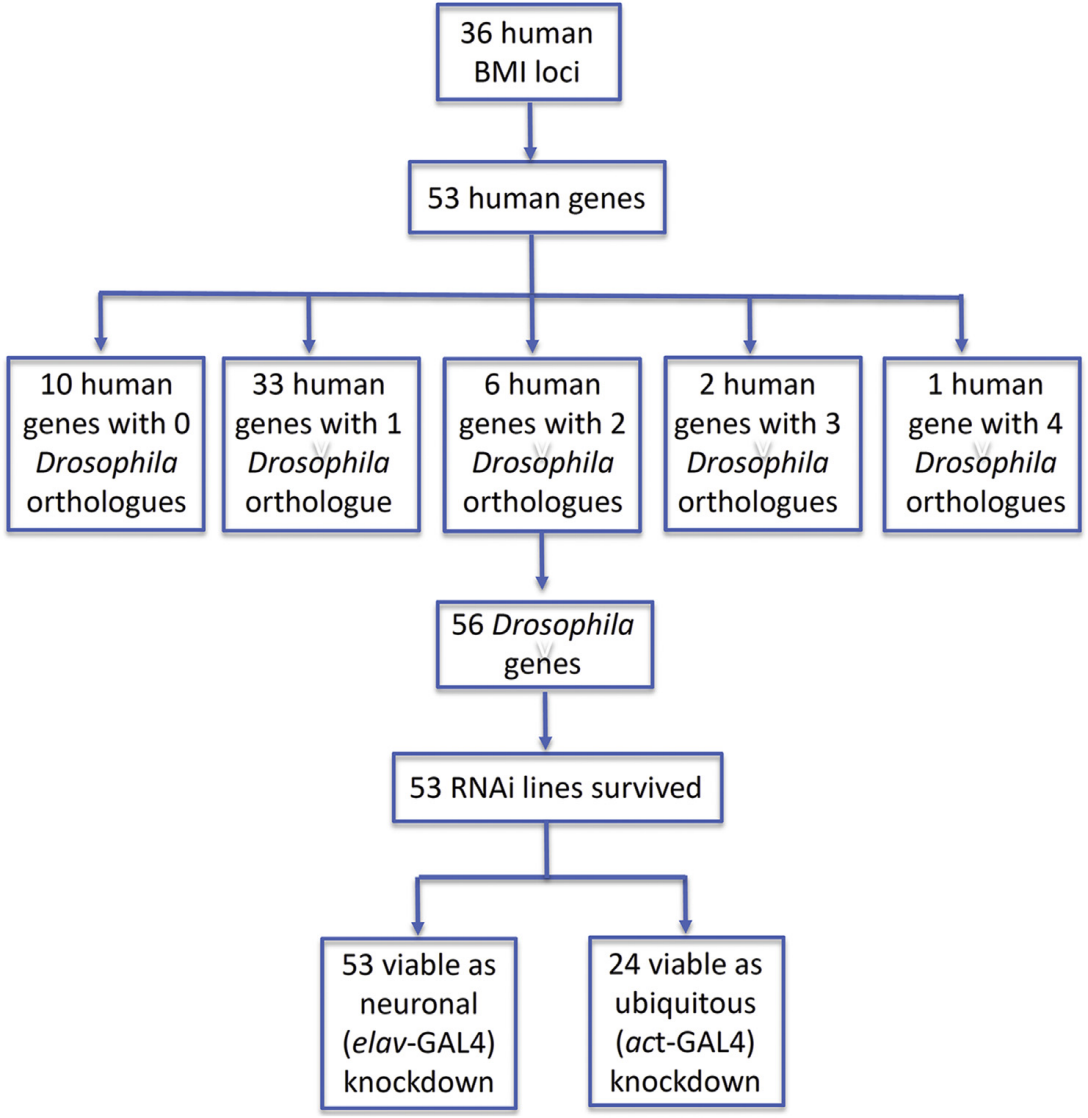
Selection of BMI GWAS genes.

Having validated our assays, we used them to screen candidate genes associated with BMI by GWAS. We focussed on 36 genetic loci identified by Speliotes et al. [49] and Lu et al. [50]. We studied the closest gene to each of the 36 BMI-associated SNPs (Supplementary Table 2), as well as additional genes within 500 kb of 7 of the SNPs, for a total of 53 human genes (Supplementary Tables 2 and 3). Forty-two of the human genes (81%) had at least 1 *Drosophila* orthologue and some had more than 1, giving a total of 56 *Drosophila* genes (Figure 2). *UAS-RNAi* lines were readily available for 53 of these genes.

Of the 56 *Drosophila* genes selected for our screen, 55 are expressed in the *Drosophila* brain [[51], [52], [53]], suggesting conservation of expression. *CG10920*/*MTCH2* is not neuronally expressed but is expressed in the fat body and should therefore be unaffected by neuronal RNAi.

**Figure 3:**
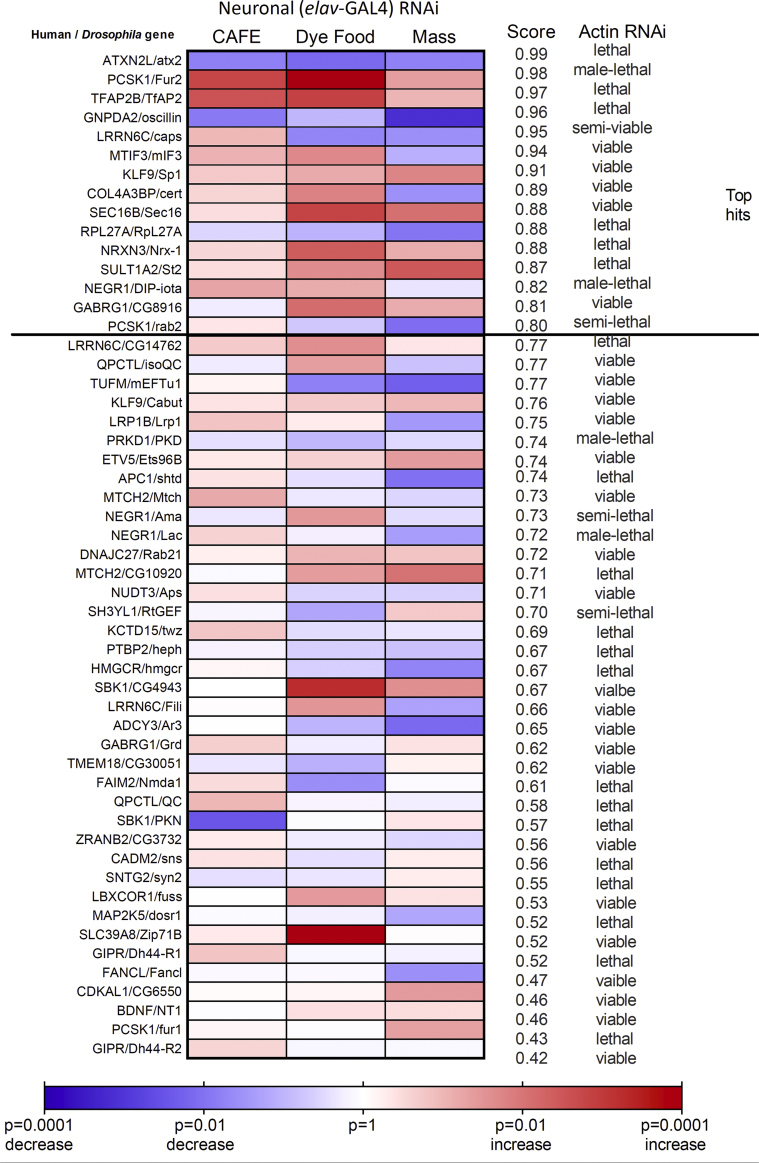
Neuronal (*elav-*GAL4) knockdown of BMI GWAS genes.

Ubiquitous RNAi knockdowns (using the *act5C-GAL4* driver) of 29 of the 53 lines tested (55%) were not viable as adult flies (Figure 3). This was much higher than the *Drosophila* genome-wide figure of 25% [36], suggesting that the BMI-associated GWAS genes are enriched for those that are essential for life.

In contrast with the ubiquitous knockdowns, neuron-specific knockdown (using *elav-GAL4*) of all 53 genes produced viable adults. Neuronal knockdown of 15 fly genes, corresponding to 14 human genes, resulted in a phenotype score of 0.80 or more in our assays (Figure 3). Several of these genes have murine orthologues with known roles in energy homeostasis (*ATXN2L, PCSK1, NEGR1, MTIF3, SEC16B,* and *TFAP2B*), increasing our confidence that our screen can reveal biologically relevant candidates. Other genes (*GNPDA2, NRXN3, GABRG1, LRRN6C, KLF9*, and *SULT1A2*) are relatively unexplored in relation to energy homeostasis, marking them prime candidates for further study.

At 7 of the GWAS loci, multiple human genes were studied in our screen (Supplementary Table 3). At 3 of these loci, the nearest gene to the SNP resulted in the highest score of all the other genes in the vicinity: *GNPDA2 (0.96), DNAJC27* (0.72), and *QPCTL (0.77)*. At 3 other loci, the highest score was observed by neuronal knockdown of ‘non-nearest’ genes: *ATXN2L* (0.99), *COL4A3BP* (0.89), and *APC1* (0.74). In addition, at the rs16951275 locus, neither the nearest gene (*LBXCOR1*) nor the nearby gene (*MAP2K5*) had a score >0.70, perhaps implicating other genes farther away than 500 kb.

Baranski and colleagues [54] performed a screen of triglyceride levels in *Drosophila*, in which BMI GWAS candidate genes were concurrently knocked down in the CNS and fat body (using the cg-GAL4 driver). Sixteen *Drosophila* genes were studied in their screen and ours (Supplementary Table 4). In their screen, 5 of these genes showed an increase in triglycerides when knocked down. In our screen, 3 of these, namely *NRXN3*, *SEC16B*, and *COL4A3BP*, had scores >0.80 (Figure 3; Supplementary Table 4). Notably, *Atx2* (corresponding to the human gene *ATXN2L*) showed no phenotype in the Baranski et al. triglyceride screen but achieved the highest score of 0.99 in our screen. However, in our hands, the knockdown of *Atx2* resulted in flies that ate less, and mice without *Atxn2* show a phenotype of adult-onset obesity [55]. Thus, although perturbation of its expression resulted in opposite feeding phenotypes in flies and mice, *Atx2* and *Atxn2* play a role in energy balance [56].

Notably, when Baranski and colleagues knocked down *RPL27A* in the CNS and fat body, the result was a lethal phenotype. In our hands, neuron-specific manipulation of *RPL27A* expression resulted in a score of 0.88, implicating *RPL27A* in the control of food intake.

### 3.4 Follow-up studies in larvae and adult knockout lines

**Fig S2:**
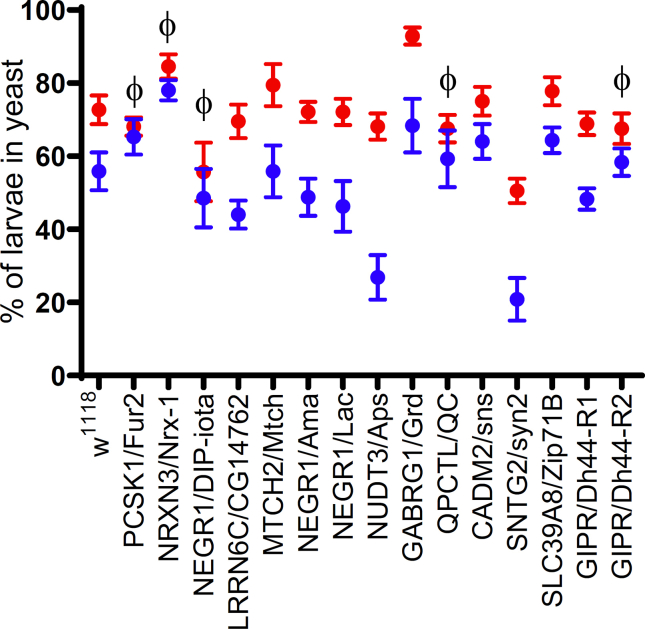


Next, we followed up the results of the neuronal RNAi experiments by studying *Drosophila* third-instar larvae with genetic knockout of the same genes [57] as an orthogonal biological replication. Knockout lines were available for 38 of the 56 genes tested, of which 15 were homozygous viable. We used an assay that measured feeding behaviour after a fast. Larvae were either fed on liquid yeast or fasted (provided with distilled water) for 2 h. Twenty larvae from each group were then transferred to the inside rim of a plate containing apple juice agar with yeast paste placed at its centre, and the numbers of larvae inside and outside the food were counted after 20 min. This assay was validated by its ability to distinguish fed from fasted third-instar wild-type larvae: 56% of fed larvae were found in the food versus 73% of fasted larvae (p = 0.017; Supplementary Figure 2). Of the 15 viable lines, 5 (*PCSK1*, *NRXN3*, *NEGR1*, *QPCTL*, and *GIPR*) did not display this anticipated increase in the number of larvae found in food after a fast (Supplementary Figure 2). *PCSK1*, *NRXN3*, and *NEGR1* have been highlighted by the aforementioned neuronal RNAi studies, with composite phenotype scores >0.80 (Figure 3).

**Fig S3:**
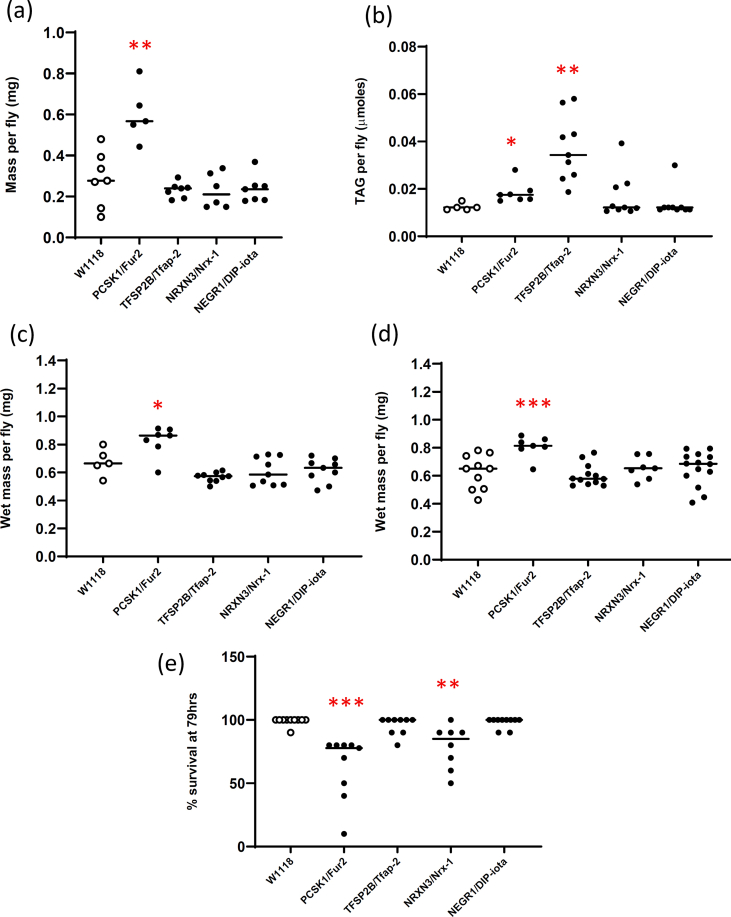


We also studied the adult *PCSK1*, *NRXN3*, and *NEGR1* knockout lines, as well as *TFAP2B*, which was also highlighted by the neuronal RNAi studies with a phenotype score of 0.97 (Figure 3). We examined dry mass, wet mass, triglyceride levels, and survival during starvation in these 4 lines and found that *PCSK1* showed a consistent ‘obesity’ phenotype (Supplementary Figure 3), in accord with the involvement of *PCSK* in human monogenic obesity [18]. In human and rodent data, mutations affecting expression of *PCSK1*result in an increase in body weight and food intake [24,28,30].

### 3.5 Screening of genes reciprocally regulated in mammalian POMC and AgRP neurons

**Figure 4:**
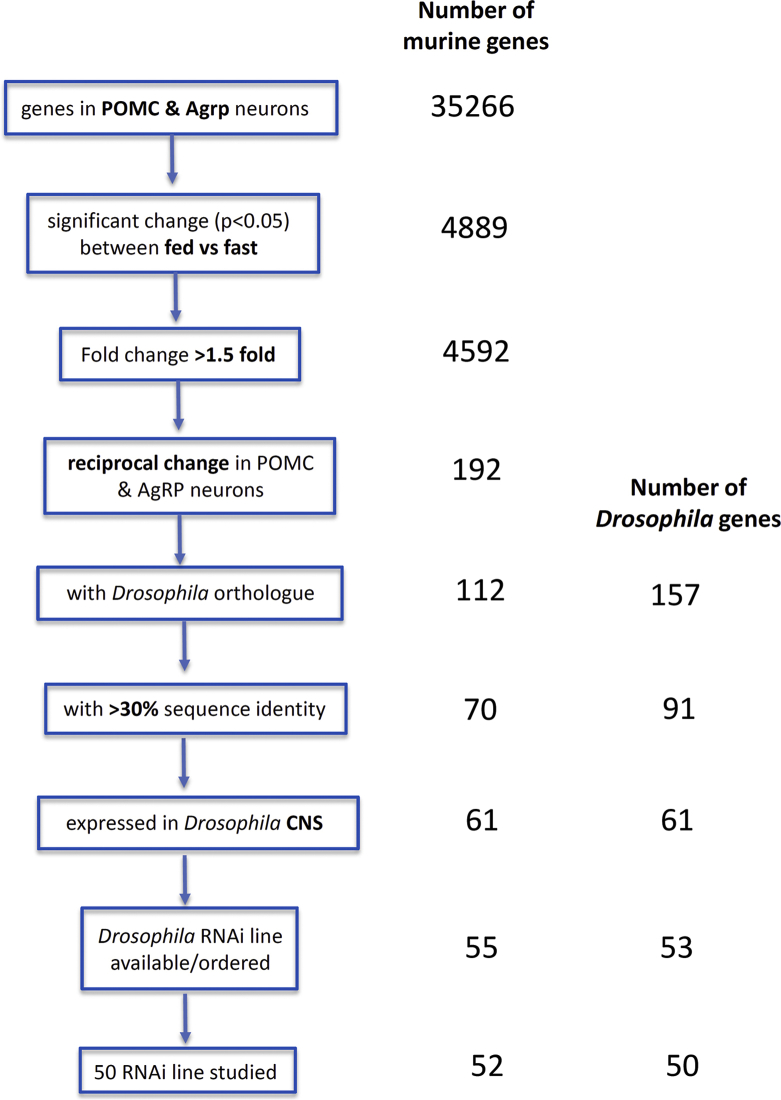
Selection of genes reciprocally regulated in murine POMC and AgRP neurons in response to fasting.

**Figure 5:**
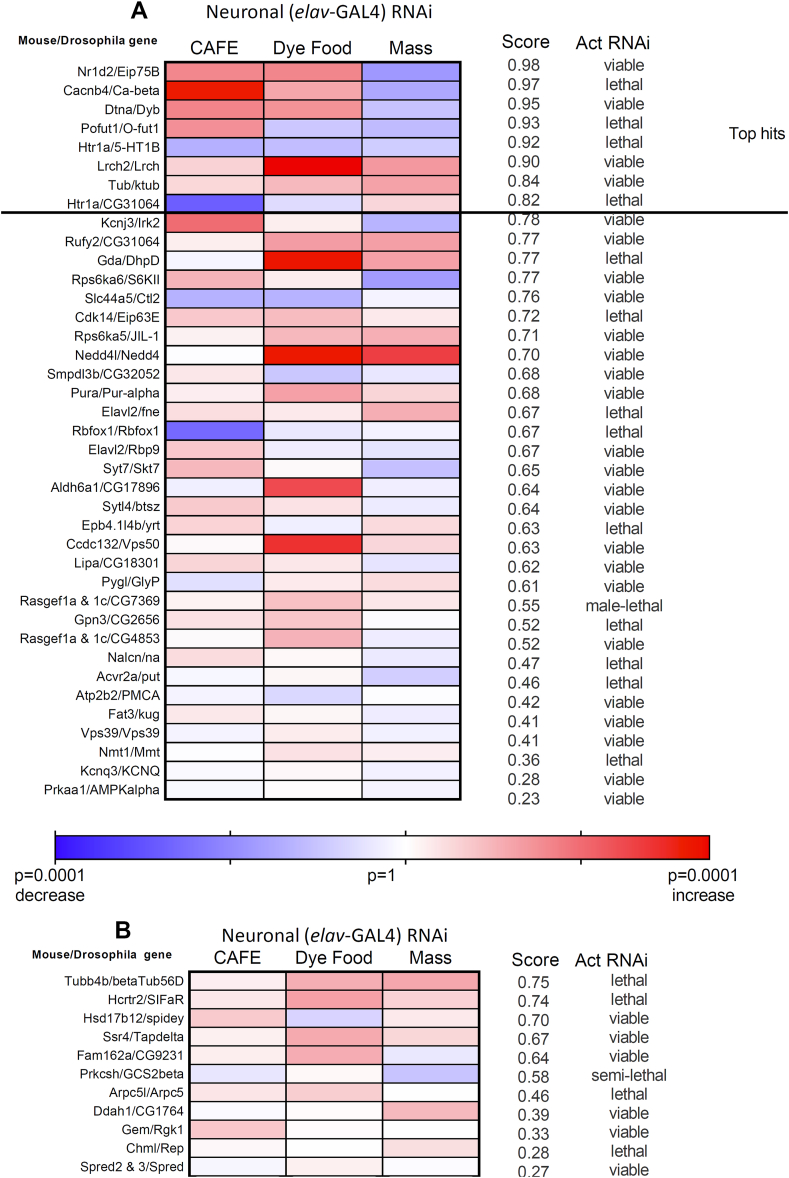
RNAi screen of murine “anorexigenic” and “orexigenic” genes. Summary of phenotypes, ranked by overall score, of flies with neuronal RNAi knockdown (using *elav*-GAL4) of the murine anorexigenic genes (a) and orexigenic genes (b). The top hit genes are those with score ≥0.80. The viability of flies when ubiquitous knockdown of the gene (using *actin-GAL4*) was performed are shown in the far right column.

Next, we assessed candidate genes emerging from transcriptomic analyses of neurons with key roles in the control of mammalian appetitive behaviour. Henry et al. [21] reported the expression levels of 35,266 mouse genes in POMC and AgRP neurons. We used a workflow to select a list of candidate transcripts with potential relevance to feeding, from this dataset (Figure 4). Of the 1,038 and 3,554 genes whose expression significantly changes (>1.5×; p < 0.05) in POMC and AgRP neurons, respectively, upon fasting, 192 responded in a reciprocal manner in the 2 neuron types. Using ENSEMBL, we identified *Drosophila* orthologues for 58% of these mouse genes, including some with multiple orthologues, and 2 mouse genes with the same fly orthologue. Overall, this resulted in a list of 157 *Drosophila* genes that corresponded to 112 murine genes. To maximise the likelihood of generating relevant biological insights, we selected orthologues of 61 mouse genes with amino-acid identity >30% and expressed in either larval or adult *Drosophila* CNS [51] (Figure 4). We again identified *Drosophila UAS-RNAi* lines for these genes, and after excluding 2 genes with no available *UAS-RNAi* line, and 6 for which only lines with multiple off-target effects were available, we screened the remainder for the effects of neuronal knockdown. In total, we assayed 11 ‘orexigenic’ genes (decreased expression in POMC neurons and increased expression in AgRP neurons during a fast) and 39 ‘anorexigenic’ genes (increased expression in POMC and decreased expression in AgRP neurons during fasting; Figure 5; Supplementary Table 5).

**Fig S4:**
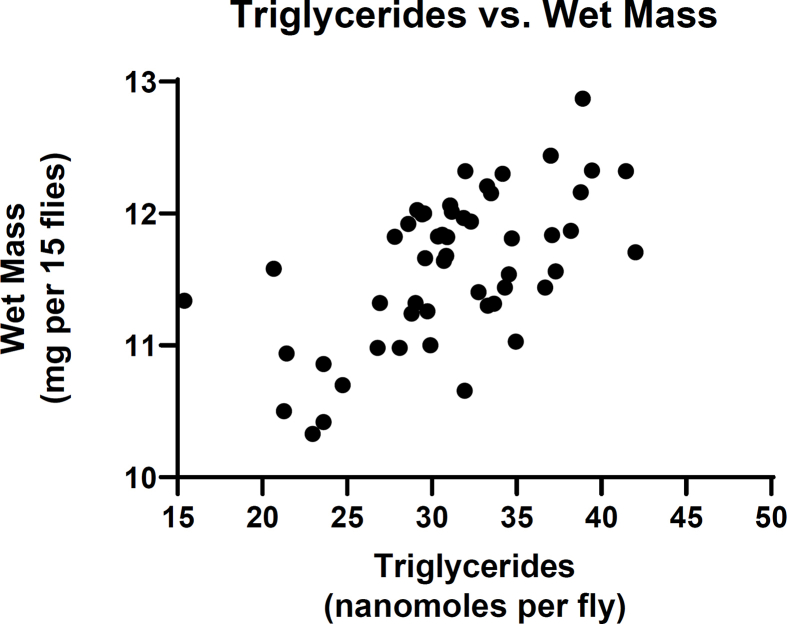


We also measured triglyceride levels in these lines and demonstrated that the values correlated well with wet mass (Pearson r = 0.6, p < 0.0001; Supplementary Figure 4). When we then used the triglyceride levels instead of wet mass in our screen, the ranking of the overall selected candidate gene list remained similar to either measurement (Supplementary Table 6). Because it is cheaper and quicker to measure mass than triglyceride levels, we suggest that wet mass is a suitable proxy for triglyceride levels in the context of a high-throughput screen.

The results of the screen, with genes ranked by score, are presented in Figure 5. The phenotype scores had a larger range (0.23–0.98) than the GWAS genes did. Seven of the ‘anorexigenic’ genes (*Nrld2*, *Cacnb4, Dtna, Pofut1, Htr1a, Lrch2,* and *Tub*) and none of the candidate ‘orexigenic’ gene had a score ≥0.80. For 2 of the genes with a score >0.80 (*Htr1a*, *Tub*) and 3 of the genes with a score between 0.70 and 0.80 (*RPS6ka5, Nedd4l, and Rufy2*), evidence is available from mouse and/or human studies for roles in energy homeostasis.

## 4 Discussion

With the rapid proliferation of gene lists emerging from large-scale ‘omics’ approaches, the aim of this study was to develop an *in vivo* high-throughput screen of feeding behaviour and energy balance phenotypes by using *Drosophila* as a model. We identified key characteristics that would make such a screen effective for prioritising long lists of gene candidates for more detailed and resource-intensive follow-up studies: examines multiple phenotypes and is economical in time and cost and simple to perform. Together, these factors allow the screen to be deployed at scale. We screened approximately 60 different *Drosophila* lines in 5 weeks, at a cost of less than £50 per gene, resulting in 30% of human genes screened being highlighted as warranting further study.

Prior *Drosophila* screens have focused on single metabolic measures in either adult flies [54,[58], [59], [60]] or larvae [61]. By contrast, we pulled together 3 assays that measured food intake and body weight, all of which fulfilled the requirements of low cost, speed, and hence, scalability. Using multiple assays and generating an integrated score offers 2 major advantages over single-assay screens. First, the screen is less likely to produce false positive results. Second, the sensitivity of the screen is increased, highlighting genes that do not show a strong phenotype in any single assay but that have subtle changes in multiple assays. Notably, this approach could possibly miss genes that affect single parameters. For example, SBK1/CG4943 and SLC39A8/Zip71B might be involved in the starvation-induced feeding regulation, as suggested from their significant effect on the fasting-induced overeating assay, but not on normal food intake and body weight (Figure 3).

While validating the screen, we initially grappled with selecting suitable negative controls. The problem is that many human diseases are associated with changes in body weight [[62], [63], [64], [65], [66], [67], [68]], as either a cause or a consequence [69], making it difficult to differentiate these genes from those that primarily influence feeding behaviour. Our solution was to use 2 categories of genes—those associated with neuronal diseases [[70], [71], [72]] and those associated with peripheral diseases [[77], [76], [75], [74], [73]]and our combined assay successfully discriminated between the negative and positive controls (Figure 1H) with significantly different average scores between the 2 groups. However, because a higher score for the *unc13-4a/UNC13* is associated with amyotropic lateral sclerosis (a neurological condition commonly associated with weight loss and dysphagia), we set a high scoring threshold for a gene to be considered for further study (0.80). Nevertheless, because the principal aim of this screen was to prioritise candidate genes for follow-up, avoiding false positive results was important; hence, we used a high threshold.

After validating the screen, we successfully demonstrated its utility for studying genes associated with BMI through GWAS [14,49], as well as genes reciprocally regulated by fasting in AgRP and POMC neurons that play a key role in the central melanocortin pathway [21].

Several genes highlighted by our screen (*PCSK1, ATXN2, NEGR1, MTIF3*, and *SEC16B*) have been shown to play a role in energy homeostasis in mouse models [24,27,28,30,32], demonstrating that the screen was effective and produced biologically relevant results. Critically, we also identified genes relatively unexplored in relation to energy homeostasis, marking them as prime candidates for further study. These included *GNPDA2, NRXN3, GABRG1, LRRN6C, COL4A3BP,* and *RPL27A*. NRXN3 (Neurexin 3), for example, is a neuronal cell surface protein involved in cell recognition and cell adhesion [78], GABRG1 (GABA(A) Receptor Subunit Gamma-1) belongs to the ligand-gated ionic channel family and is predominantly expressed in the brain reward circuitry and may be implicated in addiction [79], and GNPDA2 is an enzyme that catalyses the deamination of glucosamine-6-phosphate and is critical for lipid and glucose metabolism in human adipose-derived mesenchymal stem cells [80]. Some genes, including *Nr1d2* and *Cacnb4,* showed a *Drosophila* phenotype of significantly increased feeding but decreased mass, suggesting increased metabolic demands. We are unable to explain these apparently discordant phenotypes without detailed follow-up studies, but *Nr1d2* has been demonstrated to be important to the mammalian circadian rhythm and might play a role in energy homeostasis [81].

A problem with GWAS is that the vast majority of SNPs associated with disease are located in non-coding regions, making the identification of the ‘causative’ gene(s) driving the phenotype challenging. Therefore, we used our high-throughput screen to assay multiple genes at 7 of the BMI loci, to observe if this provided insights into the potential causative gene. Of the 24 human genes studied in these 7 loci, 5 were scored as ‘positive hits’ by our screen: *GNPDA2* and *GABRG1* (near rs10938397), *COL4A3BP* (near rs2112347), *ATXN2L*, and *SULT1A2* (near rs7359397). *ATXN2L, SULT1A2,* and *COL4A3BP* are not the genes closest to their respective SNPs. This result is comparable to those reported by Baranski, in which knocking down the nearest genes did not necessarily provide the largest phenotype.

The advantages of multiple assays over a single assay were highlighted when we compared our results to those of Baranski and colleagues, who also studied BMI-associated GWAS hits by knocking them down in *Drosophila* [54]. Sixteen genes were included in both studies, 5 of which were identified by Baranski and colleagues to have increased triglycerides storage when knocked down in the brain and fat body. Three of these 5 genes, *NRXN3*, *SEC16B*, and *COL4A3BP*, were positively identified by our screen, with an integrated score >0.80, providing independent validation of the utility of our approach. Crucially, *ATXN2L,* which when knocked down did not result in increased triglyceride storage, was positive when using our screen, because of its consistent anorexigenic effect on food intake. Atxn2 plays a role in the metabolism of branched-chain and other amino acids, metabolism of fatty acids, and in the citric acid cycle [56], with mice lacking *Atxn2* demonstrating sensitivity to diet-induced obesity [55]. Although triglyceride levels are an important readout of nutrient status, body mass was a useful proxy for them in a high-throughput screen (Supplementary Figure 4 & Supplementary Table 6).

Another unique element of our study was the use of a neuron-specific RNAi. BMI-associated genes identified by GWAS show enriched expression in the CNS [13], and 55 of the 56 genes that we studied were expressed in the *Drosophila* CNS. When knocked down ubiquitously, 55% of the lines were lethal. This is a higher proportion of lethality than for knockdown of fasting-related genes (38%) and the *Drosophila* genome-wide figure of 25% [36], suggesting that the GWAS-identified genes are essential for life. Even the Baranski study, which perturbed gene expression in both the brain and fat body, yielded 13% lethality. By contrast, all our neuron-specific knockdown lines were viable. Our more tissue-targeted approach therefore allowed us to screen more genes and obtain more phenotypic information.

In our quest for speed and scalability in this screen, we compromised on our selection of control lines: instead of testing *UAS-RNAi* lines as controls, we used the relevant *GAL4* line. However, the use of multiple *UAS-RNAi* lines from each collection should limit the extent of line-to-line variation, and other *Drosophila* screens for GWAS genes have applied a similar approach (e.g. Baranski et al. [54] and Pendse et al. [59]); thus, there is precedent. The use of multiple background controls, as well as additional confirmation of the findings using full knockout lines (as we did in some of our positive hits here), would be essential before taking a candidate gene further into a mammalian model.

One limitation of working with *Drosophila* is their distant relation to humans. However, energy homeostasis and feeding are fundamental processes for all organisms and much of the circuitry and pathways are conserved between species. Notably, nearly 80% of the genes we studied here had a *Drosophila* orthologue. Even for genes with no obvious *Drosophila* orthologue, the expression of the human gene in question can sometimes transfer the function of the encoded protein, at least in part, to the fly [82].

In conclusion, we have demonstrated that the use of *Drosophila* for screening feeding behaviour phenotypes is effective in moving from large lists obtained from whole genome or transcriptomic approaches, to more selective lists of relevant genes which can then be studied in ‘lower throughput’ and more time-consuming mammalian models. Furthermore, the detrimental effects observed in many of the ubiquitous versus neuron-specific *Drosophila* knockdowns suggests that at least for a subset of candidate genes, neuron-specific models may also be necessary to explore genotype–phenotype relationships relevant to energy balance in higher organisms.

## Funding

Initial data supported by the 10.13039/100014669BSN project support grant (Y.C.L.T). J.C was funded by a 10.13039/100004440Wellcome Trust 4-year PhD studentship. Y.C.L.T. and G.S.H.Y. are supported by the 10.13039/501100000265Medical Research Council (MRC Metabolic Diseases Unit (MC_UU_00014/1)). S.O’R is supported by the 10.13039/100004440Wellcome Trust (WT 095515/Z/11/Z), the 10.13039/501100000265MRC Metabolic Disease Unit (MC_UU_00014/1).

## Author contribution

Chalmers J: Methodology, Investigation, Formal analysis, Visualisation, Project administration, Funding acquisition. Tung YCL: Conceptualisation, Methodology, Validation, Visualisation, Supervision, Funding acquisition, Writing–Original draft preparation and editing. Liu C: Methodology, Resources, Supervision. O'Kane CJ: Methodology, Supervision, Writing–Reviewing and Editing. O'Rahilly: Conceptualisation, Writing–Reviewing and Editing, Funding acquisition. Yeo GSH: Conceptualisation, Supervision, Writing–Reviewing and Editing, Funding acquisition.

## Figures and Tables

**Table S4:**
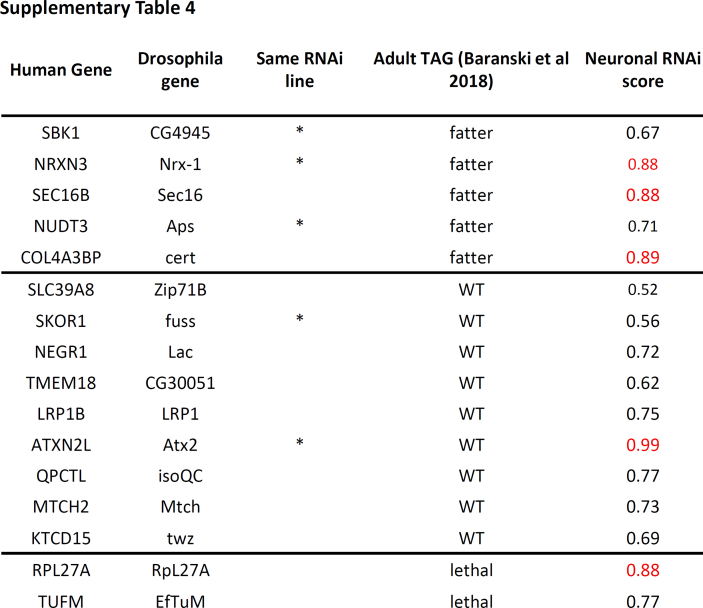


**Table S5:**
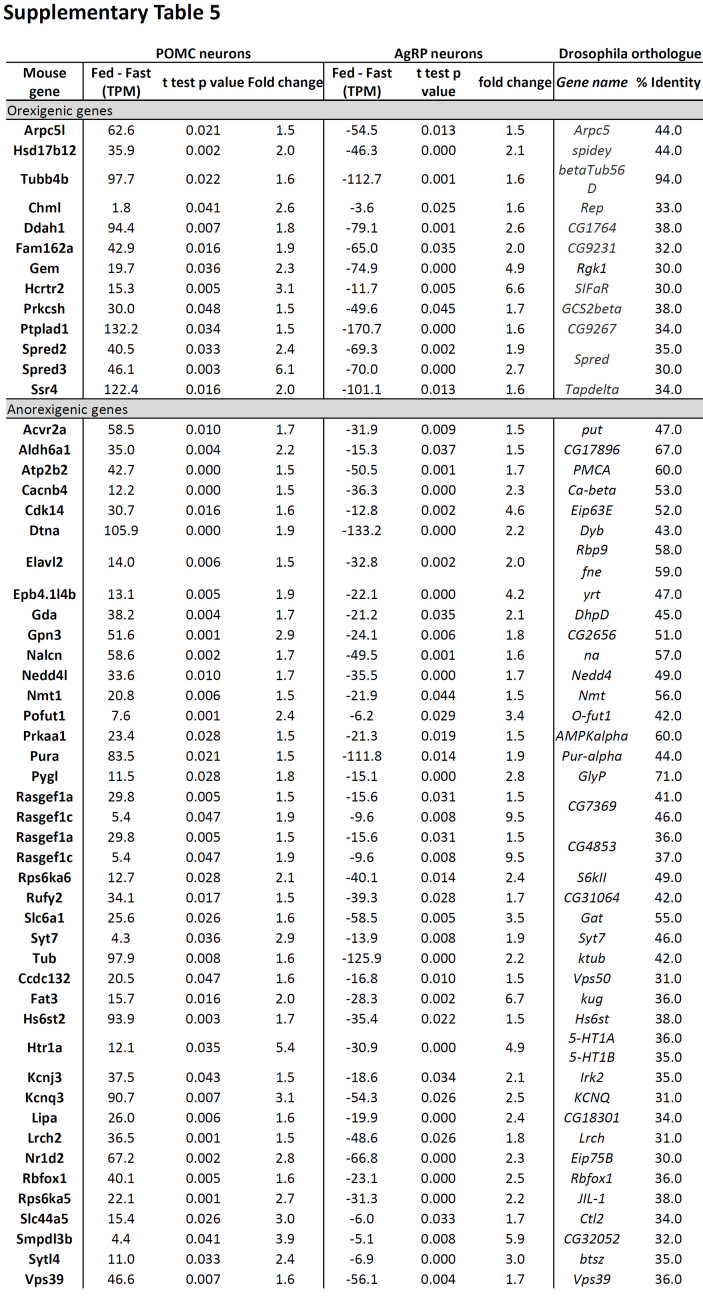


**Table S6:**
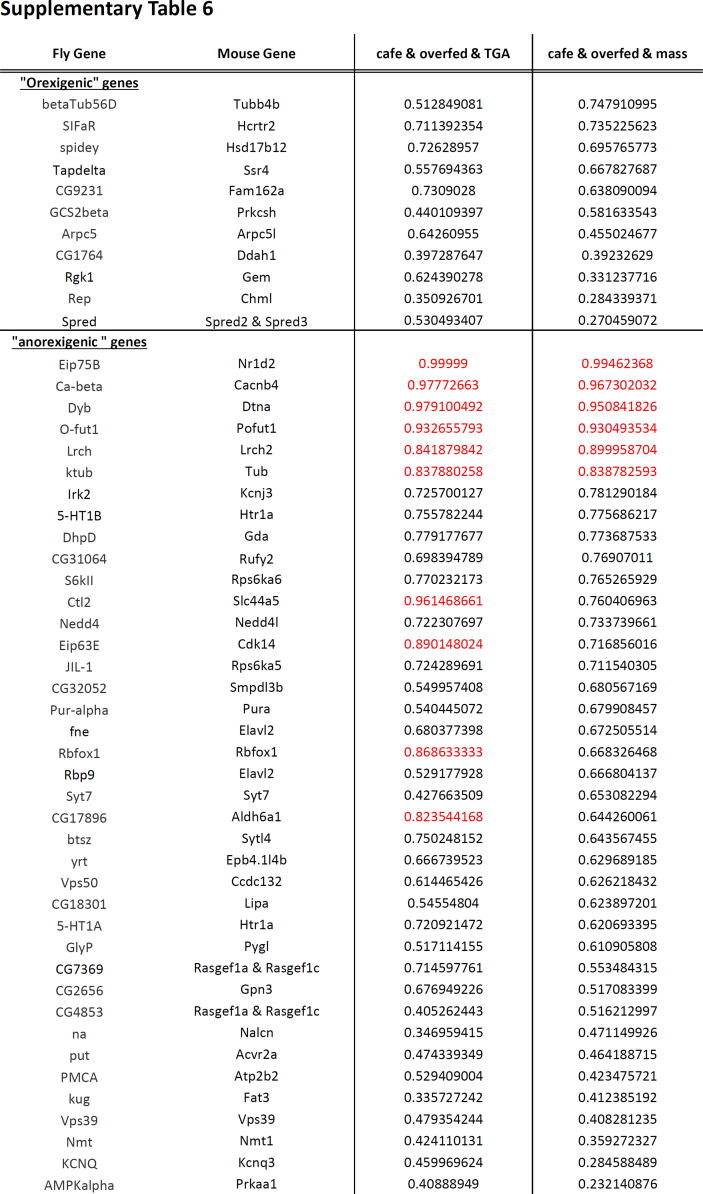


**Table S7a:**
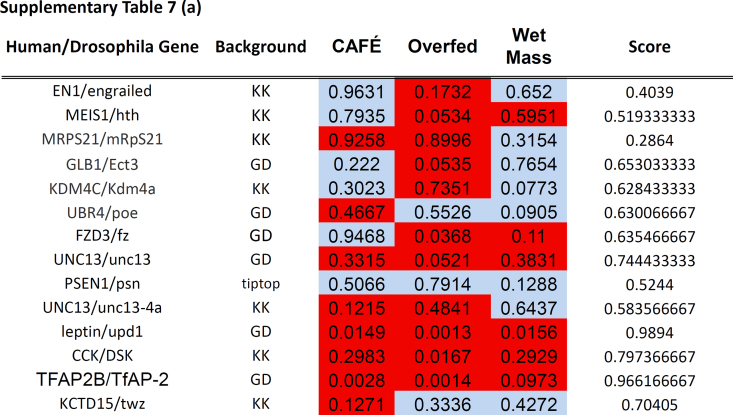


**Table S7b:**
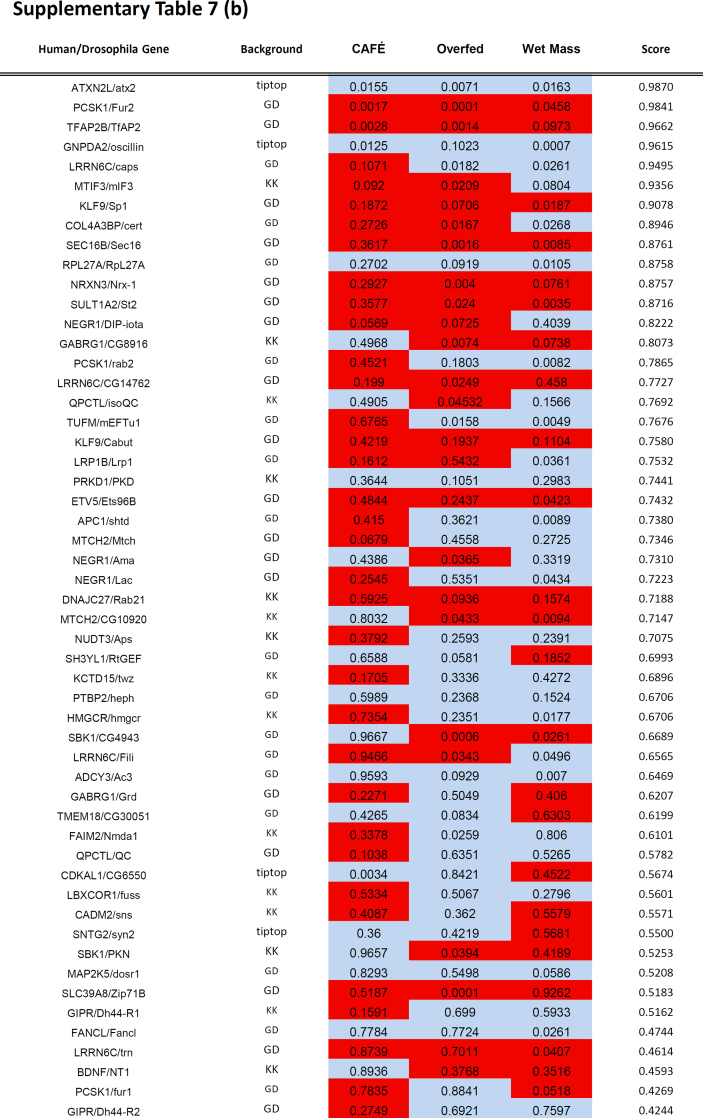


**Table S7c:**
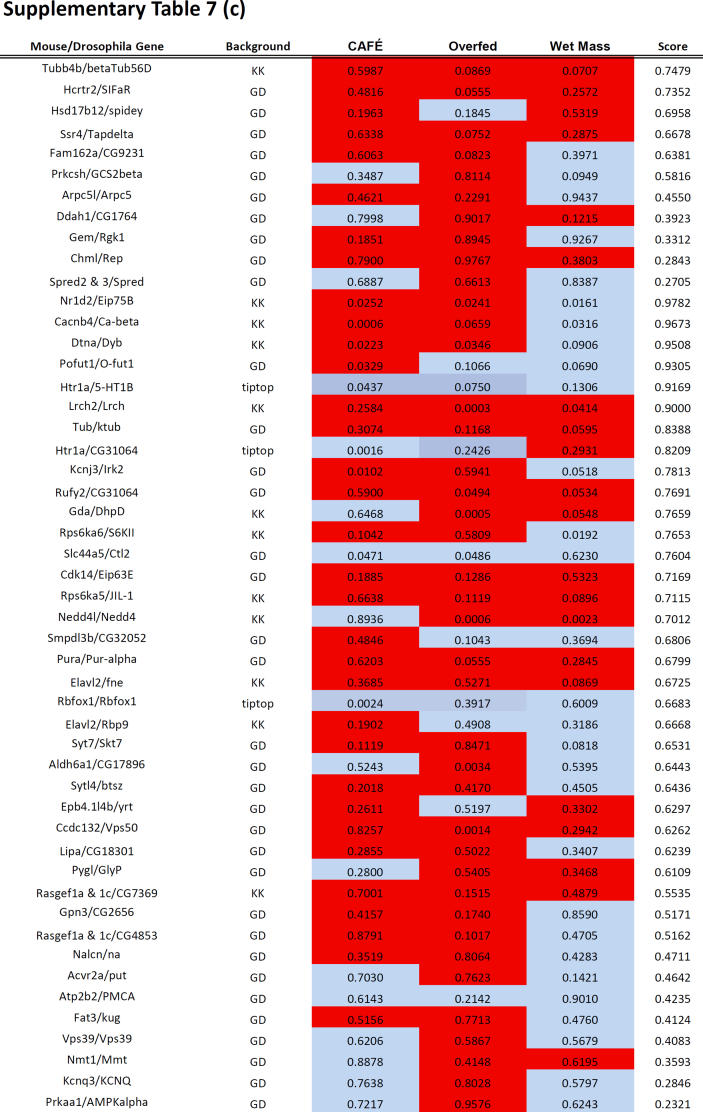


**Table S1a:**
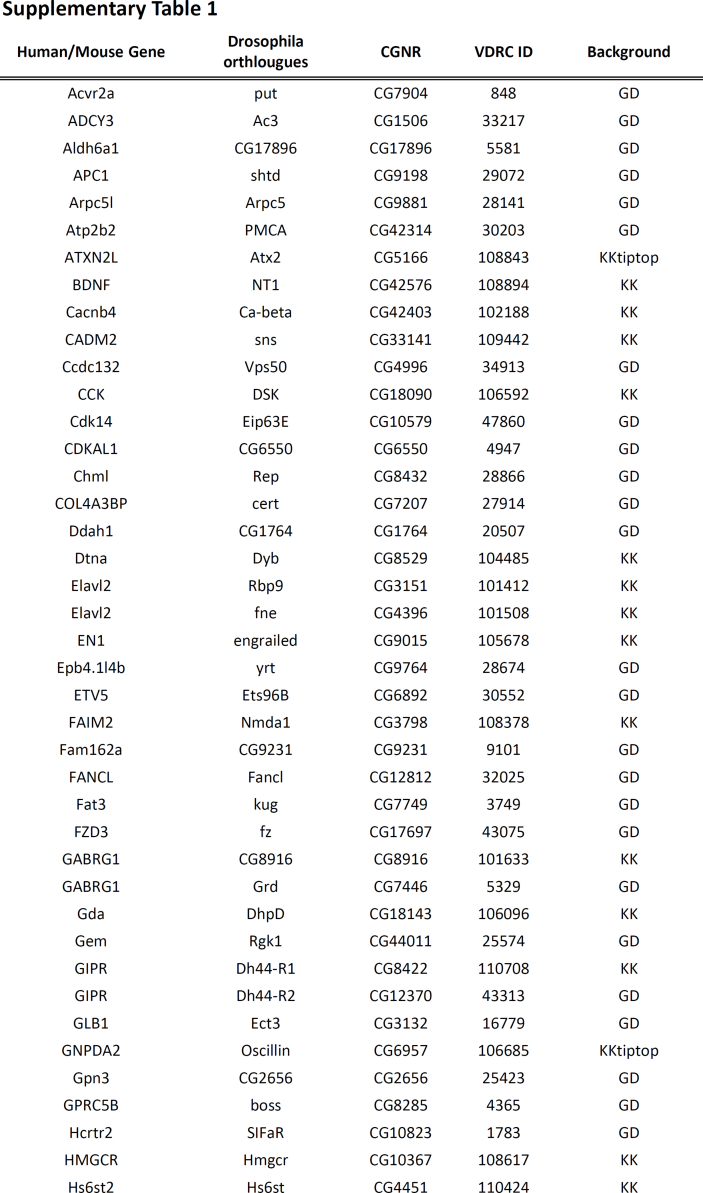


**Table S1b:**
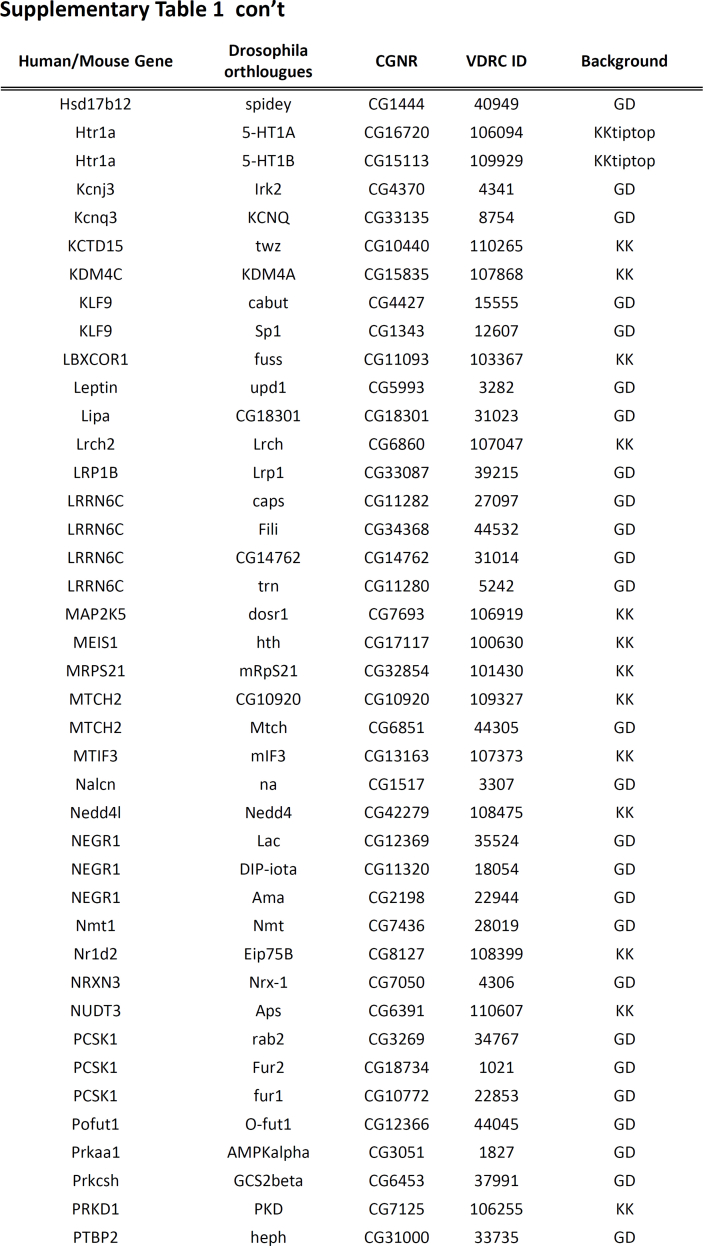


**Table S1c:**
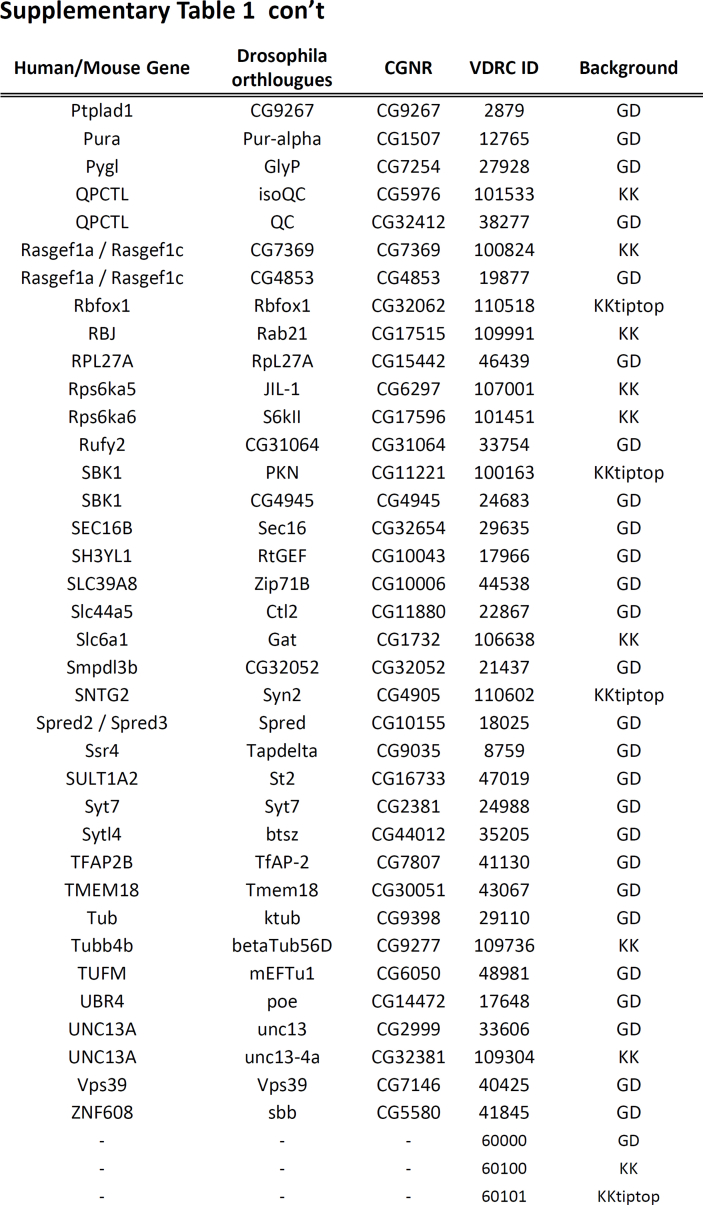


**Table S2:**
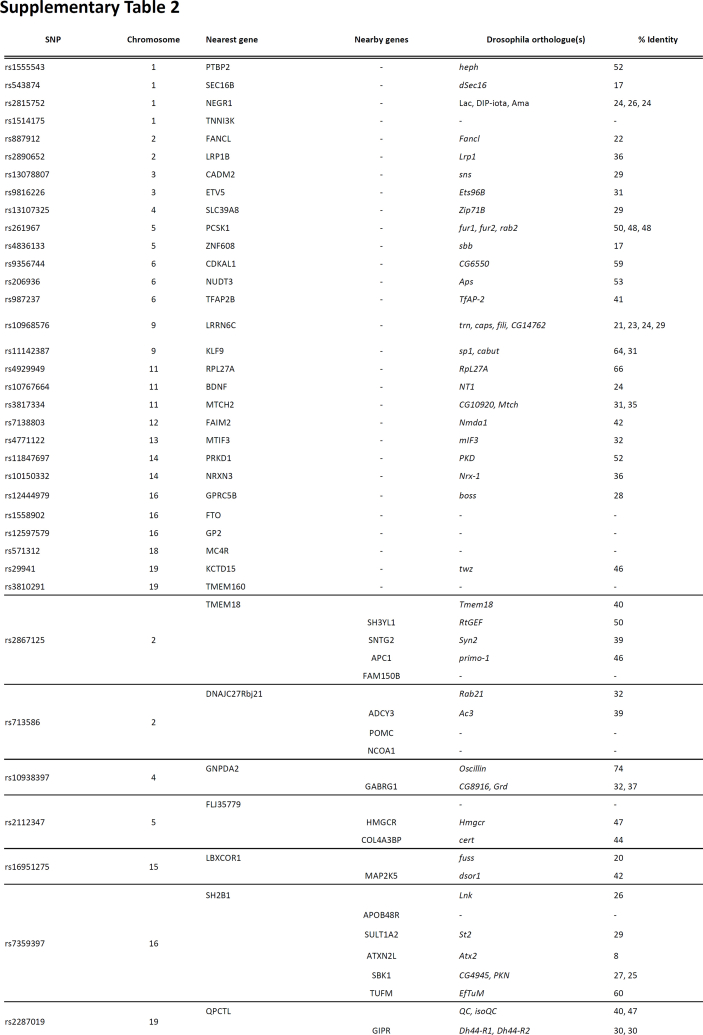


**Table S3:**